# The brain-body circuit mediates acute stress–induced antiinflammatory reflex in bacterial cystitis by suppressing ILC2 activation

**DOI:** 10.1172/jci.insight.189362

**Published:** 2025-03-18

**Authors:** Yaxiao Liu, Jinhua Wang, Junyang Lin, Dingqi Sun, Kejia Zhu, Tongxiang Diao, Qiang Fu, Qingyu Ren

**Affiliations:** 1 Department of Urology, Shandong Provincial Hospital Affiliated to Shandong First Medical University, Jinan, China.; 2 Department of Radiotherapy, Shandong Second Provincial General Hospital, Jinan, China.; 3 Department of Anatomy and Neurobiology, School of Basic Medical Sciences, Shandong University, Jinan, China.

**Keywords:** Infectious disease, Inflammation, UTI/pyelonephritis, Urology

## Abstract

Urinary tract infections (UTIs) are one of the most commonly encountered infections in clinical practice, in which psychological stress is a critical pathological contributor to modulate immune function. However, mechanistic pathways linking stress networks in the brain to bladder infection remain poorly understood. In this study, we discovered that acute stress treatment suppressed bladder inflammation in mice with UTIs, and a substantial number of neurons showing overlap between inflammation-associated markers and retrograde labeling were observed in the paraventricular nucleus (PVN) brain region of these mice. Activation of the PVN alleviated uropathogenic *Escherichia coli*–induced bladder inflammatory response. Moreover, a blocked hypothalamic-pituitary-adrenal axis reversed the antiinflammatory reflex mediated by acute stress, suggesting that glucocorticoids may modulate UTIs through the brain-body circuit. Single-cell RNA-Seq of bladder immune cells revealed that type 2 innate lymphoid (ILC2) cells expressed abundant levels of glucocorticoid receptor. The activation of the PVN effectively inhibited the expression of the pro-inflammatory cytokine colony-stimulating factor 2 by ILC2 cells through direct regulation of cell-intrinsic glucocorticoid signaling. Ultimately, our study has implications for the positioning of the brain-body circuit for UTI treatment.

## Abstract 2

Acute stress can modulate urinary tract infections through activation of the hypothalamus and then direct regulation of glucocorticoid signaling in ILC2 cells.

## Graphical abstract

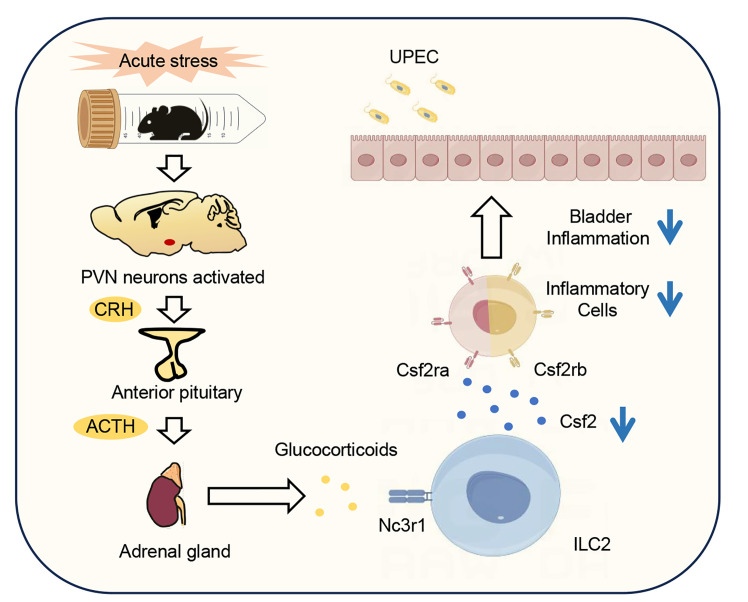

## Introduction

Urinary tract infections (UTIs) are among the most common microbial diseases, infecting over 150 million people annually and imposing a substantial financial burden on society (1). Most UTIs are caused by uropathogenic *Escherichia coli* (UPEC). Bacterial infections trigger robust host responses in the bladder, leading to the release of potent cytokines and the recruitment of extensive neutrophils and macrophages (2, 3). However, given the overuse of antibiotics and the growing threat of antimicrobial resistance, identifying alternative treatments for UTIs has become a top priority.

The central nervous system (CNS) is the central regulator of the body and receives immune-related information via sensory inputs from peripheral immune organs (4, 5). For instance, neuroimaging studies have identified increased activation in specific brain regions during peripheral inflammation. Upon receiving immune-related information, the CNS primarily responds to peripheral immune challenges through the autonomic nervous and neuroendocrine systems (6, 7). During this process, psychological stress can modulate peripheral immunity via the CNS (8). Acute stress can enhance immune function in the short term, aiding in the response to immediate threats. In contrast, chronic stress may lead to immune dysfunction or the development of diseases. Recently, it has been reported that stress-responsive brain regions innervate immunologically relevant organs, including the liver, spleen, gastrointestinal tract, and bone marrow, primarily via the autonomic nervous system and the hypothalamic-pituitary-adrenal (HPA) axis (9, 10). The activation of the HPA axis and subsequent release of glucocorticoid hormones are key responses that coordinate stress and neuroendocrine functions. The hypothalamus, including the paraventricular nucleus (PVN) (11), lateral hypothalamus (LH) (12), arcuate nucleus (ARH) (13), and more, is activated by acute stress in rodents, with the PVN being a key component of the HPA axis (14). Upon exposure to a stressor, PVN neurons that express corticotropin-releasing hormone (CRH) and vasopressin initiate the HPA axis cascade, leading to the glucocorticoids’ release by the adrenal gland (14). The release of glucocorticoids into the bloodstream effectively suppresses immune responses to limit immunopathology and mobilize energy for other activities, so it may be a potential effective treatment strategy for bacterial infections. Nevertheless, our understanding of neuroimmune interactions remains limited. Additionally, the specific neurons involved in regulating the immune system during infections, including UTIs, need further clarification.

The innate lymphoid cell (ILC) family consists of natural killer, ILC1, ILC2, and ILC3 cells, playing essential roles in the immune response against viruses, bacteria, parasites, and cancer cells (15). ILC2, distinguished by the expression of the transcription factor GATA-binding protein 3 and the production of type 2 cytokines such as Il5, Il13, and colony-stimulating factor 2 (Csf2), play a pivotal role in modulating immunity during homeostasis and pathogen challenge (6, 7). In a previous study, we found the role of bladder ILC2 contributes to the exacerbation of bladder inflammation, voiding behavior, and pelvic allodynia during UPEC infection (16). It has been demonstrated in prior research that glucocorticoids inhibit a range of inflammatory responses induced by ILC2. These results raise the possibility that a similar form of regulation might exist within bladder ILC2, where glucocorticoids may exert redundancies to suppress pro-inflammatory gene expression.

In this study, we investigate whether acute stress can improve UPEC-induced UTIs and inflammation immunity challenges, while analyzing the underlying neural mechanisms. The findings uncovered a tractable brain-body mechanism that connects psychological states to host defense and possibly translates to effective clinical interventions for individuals with UTIs.

## Results

### Stress mediates UTIs’ exacerbation.

**Figure 1:**
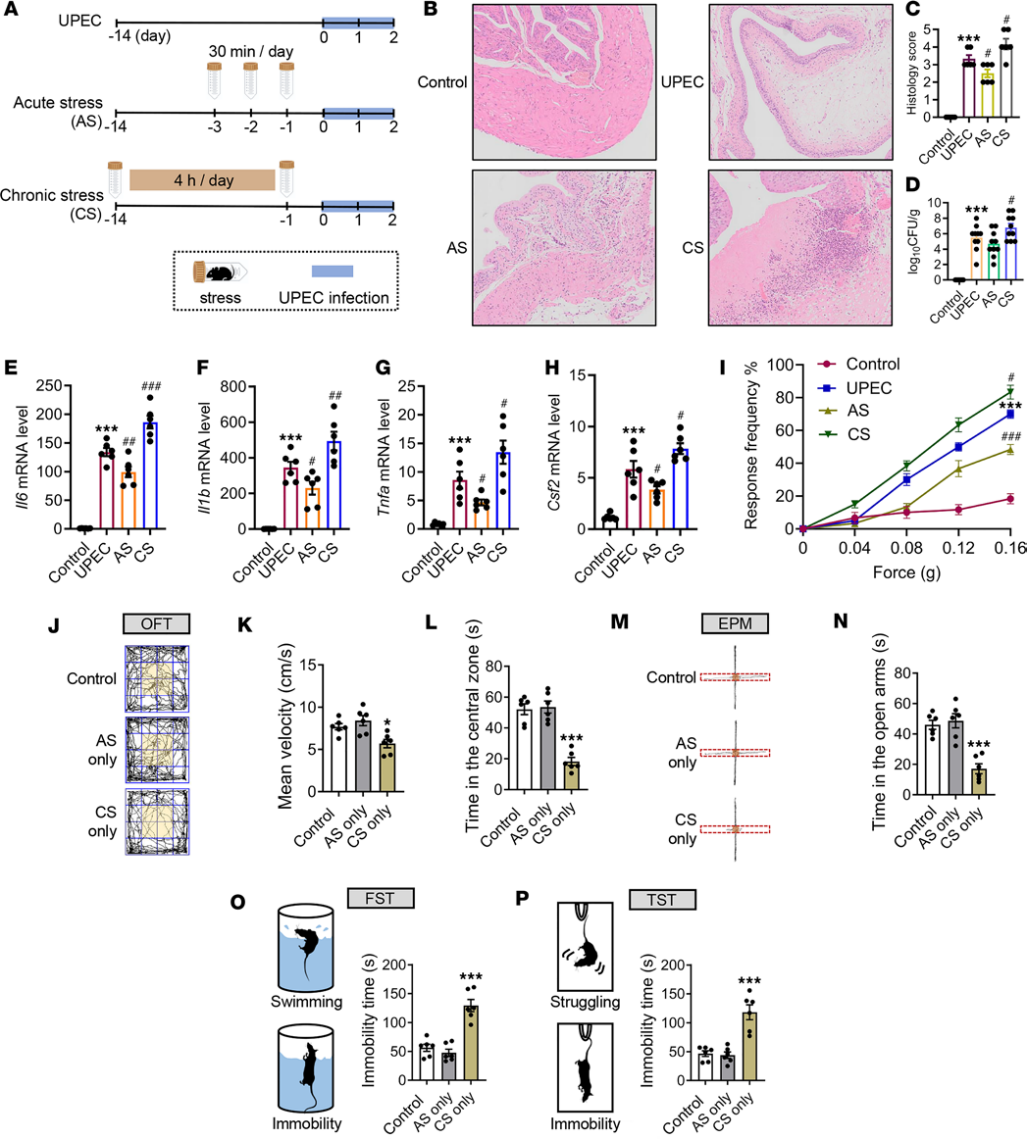
Acute and chronic stress induce divergent effects on UTI-induced bladder inflammation. (**A**) Graphical illustration of the experimental setup. Bladders were isolated from control mice, UPEC infection mice, AS-UPEC infection mice, and CS-UPEC infection mice. (**B**) H&E staining of bladders from 8-week-old female control mice, UPEC infection mice, AS-UPEC infection mice, and CS-UPEC infection mice. (**C**) Histology scores were assessed after infection (*n* = 6 mice per group). ***P < 0.001 vs. control group. ^#^*P* < 0.05 vs. UPEC group. (**D**) Bacterial load was assessed 24 hours after infection (*n* = 10). ***P < 0.001 vs. control group. ^#^*P* < 0.05 vs. UPEC group. (**E**–**H**) The mRNA levels of *Il6*, *Il1b*, *Tnfa*, and *Csf2* of mice (*n* = 6 mice per group). ***P < 0.001 vs. control group. ^#^*P* < 0.05, ^##^*P* < 0.01, ^###^*P* < 0.001 vs. UPEC group. (**I**) Mice were assessed for pelvic tactile allodynia/hyperalgesia (*n* = 10 mice per group). ***P < 0.001 vs. control group. ^#^*P* < 0.05, ^###^*P* < 0.001 vs. UPEC group. (**J**) Representative tracks of mice on the OFT. (**K** and **L**) Mean velocity in the box and time in the central area (orange area) of mice (*n* = 6 mice per group). *P < 0.05, ***P < 0.001 vs. control group. (**M**) Representative tracks of mice on the EPM. (**N**) Time in the open arms of mice (*n* = 6 mice per group). ***P < 0.001 vs. control group. (**O** and **P**) Diagram of simulation mice on the FST and TST. Immobility time of mice (*n* = 6 mice per group). ***P < 0.001 vs. control group. Results are presented as mean ± SEM and analyzed by 1-way ANOVA with Holm-Šidák corrections for multiple comparisons (**C**–**H**, **K**, **L**, and **N**–**P**) or 2-tailed Student’s *t* tests (**I**).

To investigate the mechanisms underlying the impact of stress on bladder inflammation, we utilized mouse models of acute stress (AS, 30 minutes per day for 2 days) and chronic stress (CS, 4 hours per day for 2 weeks), inducing UTIs using CFT073 (Figure 1A). Interestingly, we observed substantially different outcomes when applying restraint stress at different times during the experimental protocol. Histological changes in the bladders of mice were evaluated using H&E staining. Following UPEC infection, bladder tissues from mice in the AS group exhibited lower histology scores compared with the UPEC group. Conversely, the CS group showed substantial edema with marked inflammatory cell infiltration compared with the UPEC group (Figure 1, B and C). Compared with the UPEC group, the CS group showed an increased CFU burden, while both UPEC and AS groups had similar CFU burdens (Figure 1D). The pro-inflammatory cytokines *Il1b*, *Il6*, *Tnfa*, and *Csf2* were also substantially decreased after 48 hours from UPEC infection relative to those measured in AS mice but increased in CS mice (Figure 1, E–H). Furthermore, we assessed pain response in mice, which correlated with observed variations in bladder inflammation (Figure 1I). These results indicate that psychological stress may precondition the bladder for enhanced inflammation during subsequent encounters with a UPEC infection.

It has been reported that stress can induce anxiety and depression symptoms, contributing to disease deterioration. To determine whether acute restraint stress and chronic restraint stress increase these symptoms in mice, we conducted depression- and anxiety-related behavioral tests after restraint treatment. The results from the open field test (OFT) showed that mean velocity in box and time in central zone substantially decreased in the CS only mice compared with the control group. However, there was no difference between the AS only group and the control group (Figure 1, J–L). In the elevated plus maze test (EPM), time in open arms by the mice was not different between the control and the AS only groups. In contrast, the time spent in open arms decreased in CS only mice (Figure 1, M and N). Interestingly, in both forced swim test (FST) and tail suspension test (TST), compared with the control group, the immobility time increased in CS only mice (but not AS only mice), an indication of increased depression- and anxiety-like symptoms (Figure 1, O and P).

In summary, while the specific mechanisms require investigation in the future, these findings suggest that CS may primarily increase depression- and anxiety-like behaviors in mice, thereby exacerbating bladder inflammation. Consequently, we further examined the causes and mechanisms by which AS ameliorates UTIs as an interesting research direction.

### Increased neuronal activity in the PVN during UTIs.

**Figure 2:**
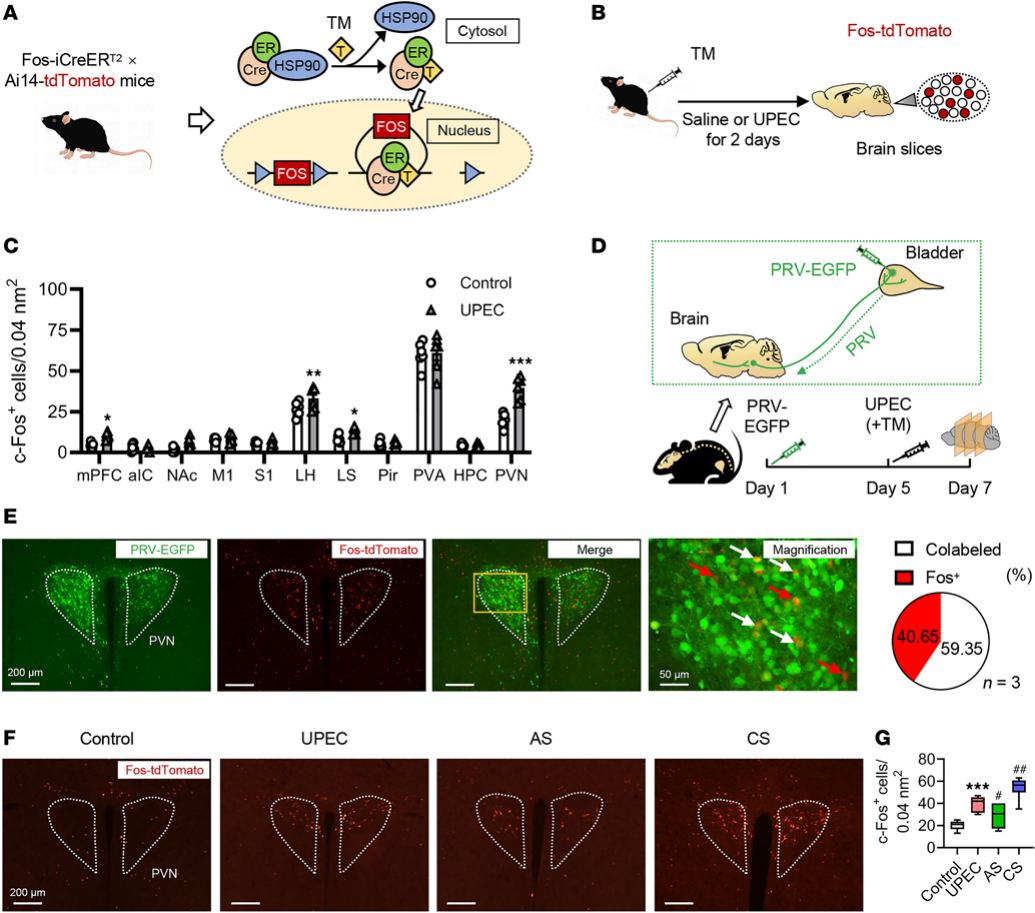
Identification of brain regions involved in bladder inflammation. (**A**) Experimental setup. Fos-iCreER^T2^ Ai14-tdTomato mice were treated with TM to label active neurons. (**B**) Experimental timeline of the bladder inflammation mouse model. (**C**) c-Fos^+^ cell numbers in several brain neurons (*n* = 6 mice per group). *P < 0.05, **P < 0.01, ***P < 0.001 vs. control group. (**D**) Schematic and experimental timeline of PRV-EGFP injection into the bladder wall. (**E**) Left: the representative image of PRV-infected (green) and TM-labeled (red) colabeled neurons in PVN. Scale bar, 200 μm. Magnified image. Scale bar, 50 μm. Right: quantification of the percentage of colabeled neurons in the Fos^+^ population averaged from *n* = 3 mice. (**F** and **G**) Representative images of c-Fos expression in PVN labeled with tdTomato (red) (*n* = 6 mice per group). ***P < 0.001 vs. control group. ^#^*P* < 0.05, ^##^*P* < 0.01 vs. UPEC group. Scale bar, 200 μm. Box plots show the interquartile range, median (line), and minimum and maximum (whiskers). Results are presented as mean ± SEM and analyzed by 2-way ANOVA with Holm-Šidák corrections for multiple comparisons (**C**) or 1-way ANOVA with Tukey’s corrections for multiple comparisons (**G**).

To capture neurons that were active during the inflammation of the bladder, we used transgenic targeted-recombination-in-active-populations (*TRAP*) mice. These mice express iCreER^T2^ under the control of an activity-dependent c-Fos promoter (*FosTRAP* mice), which serves as an indicator of neuronal activity (17). In the presence of tamoxifen (TM), active neurons drive Cre-dependent recombination to induce the expression of an effector gene (e.g., fluorescent reporter). We crossed the *TRAP* mice with a Cre-dependent tdTomato reporter line, *Ai14*, to visualize the active neuron cells (Figure 2A).

Mice were injected with TM (inducing Cre recombination and as a result, tdTomato expression) 2 hours before initiation of the UPEC treatment (UPEC group). As controls, we used another group of *TRAP*-tdTomato mice that were injected with TM at the same time but with saline (control group; Figure 2B). This group allowed us to compare between inflammation-related activity in the brain and brain activity during healthy homeostasis. After UPEC or saline treatment for 48 hours, the whole mouse brain sections were observed under Olympus fluorescence microscope. The results revealed that compared with saline group, UPEC group increased tdTomato expression (indicative of neuronal activity) in several brain areas (Figure 2C), including the medial prefrontal cortex (mPFC) (involved in cognitive, social, and reward behavior), the LH nuclei (involved in motivation and sleep-wake behavior), the lateral septum (LS) (involved in anxiety, fear, and aggressive behavior), and the PVN (neuroendocrine and autonomic nervous function regulation) (Supplemental Figure 1; supplemental material available online with this article; https://doi.org/10.1172/jci.insight.189362DS1).

To identify neurons that commonly participate in bladder control and inflammation, we first injected pseudorabies viruses (PRV) encoding EGFP directly into the bladder wall of adult male mice to retrogradely and trans-synaptically label upstream neurons. Furthermore, neurons activated during UPEC treatment were labeled (tdTomato) by TM injection on day 5 (Figure 2D). The results showed a substantial overlap between bladder control neurons (EGFP) and bladder inflammation neurons (tdTomato) in the PVN region (Figure 2E), indicating that neurons included in the *TRAP*ed ensembles in the PVN, which can induce UTIs, have a direct path of communication with the bladder. Consistent with the results of the degree of UTI, compared with the UPEC group, the excitability of PVN neurons was decreased in UTI mice after AS and increased in UTI mice after CS, which verified that the PVN was highly involved in the process of stress and UTI (Figure 2, F and G). Thus, consistent with previous studies, the brain, including the PVN, responds to peripheral immune challenge.

### PVN responses to immune cytokines.

**Figure 3:**
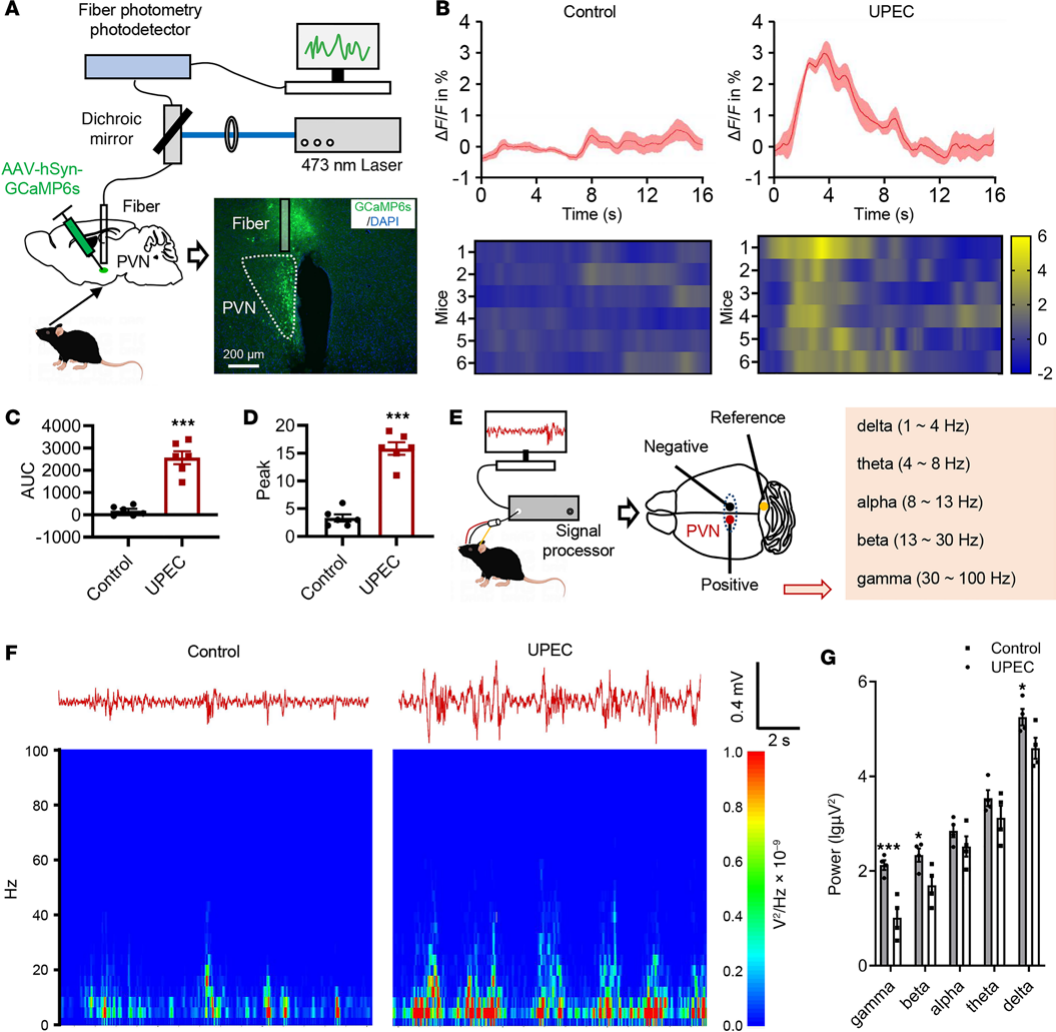
UPEC-induced bladder inflammation upregulates neuronal activity in the PVN. (**A**) Schematic diagram illustrating the recording of calcium signals in the PVN. Scale bar, 200 μm. (**B**) Upper: average calcium signals (ΔF/F in %) synchronized during bladder Inflammation or after saline treatment. Thick lines, mean; shaded areas, SEM. Lower: heatmap of calcium signals in each mouse. (**C**) The AUC of calcium signal recording in the PVN neurons (*n* = 6 mice per group). ***P < 0.001 vs. control group. (**D**) The peak of calcium signal recording in the PVN neurons (*n* = 6 mice per group). ***P < 0.001 vs. control group. (**E**) Diagram illustrating the localization of LFP electrode implants in PVN. (**F**) Upper: representative examples of LFP traces from the PVN in saline or UPEC mice. Lower: heatmap of LFP signals. (**G**) LFP power in the different frequency bands in the PVN from 4 mice per group. *P < 0.05, ***P < 0.001 vs. control group. Results are presented as mean ± SEM and analyzed by 2-tailed Student’s *t* tests (**C** and **D**) or 2-way ANOVA with Holm-Šidák corrections for multiple comparisons (**G**).

To identify the neuronal changes of PVN during bladder inflammation, we applied in vivo real-time fiber photometry and local field potential (LFP) techniques. Fiber photometry was used to detect changes in intracellular Ca^2+^ levels, to specifically monitor the neural activity of the PVN region in the presence of the GCaMP6s virus (Figure 3A). The results have shown that UPEC-treated mouse PVN neuronal activity showed an increase and maintained a plateau, whereas the saline treatment mice exhibited a much slighter increase in sensitivity, which faded away in a very short time, in the activity of PVN neurons (Figure 3B). The PVN neurons of the UPEC group mice showed a higher area under the curve (AUC) (Figure 3C) and peak (bigger responses) (Figure 3D). In addition, to investigate the effect of the micturition cycle on PVN neurons, we incorporated fiber photometry to measure the excitability of PVN neurons during bladder filling and micturition in both control and UPEC-treated mice. The results (including time stamp) revealed that PVN neurons were more active during micturition compared with bladder filling. The excitability of PVN neurons in the UPEC group was substantially higher than that in the control group during bladder filling, micturition, and immediately following micturition (Supplemental Figure 2, A–C). These results suggest that UPEC-treated bladder inflammation upregulates Ca^2+^ levels and neuronal activity in PVN neurons.

To further investigate whether the functional changes in PVN neurons were accompanied by abnormal neuronal discharge, we used a LabChart LFP detection device to investigate the effects of UPEC on neuronal oscillations in the PVN region in mice. Figure 3E shows the schematic of the LFP traces from the PVN. We observed that the delta, theta, alpha, beta, and gamma oscillations were increased in the PVN in UPEC-treated mice. Remarkably, delta oscillations have been reported to be involved in sleep, emotional, and some pathological signals (18–21). Theta oscillations have been related to active motor cognitive and behavior processes (22, 23). Alpha and beta oscillations are involved in reward events (24), while beta and gamma oscillations are involved in contextual memory (25). Our data indicated that UPEC upregulated neuronal oscillations in PVN neurons (Figure 3, F and G). Taken together, these findings strongly indicate a close relationship between PVN neurons and bladder inflammation in mice, implying that modulation of the brain/bladder axis could potentially ameliorate bladder inflammation.

### Chemogenetic activation of the PVN region attenuates UTI-induced bladder inflammation.

**Figure 4:**
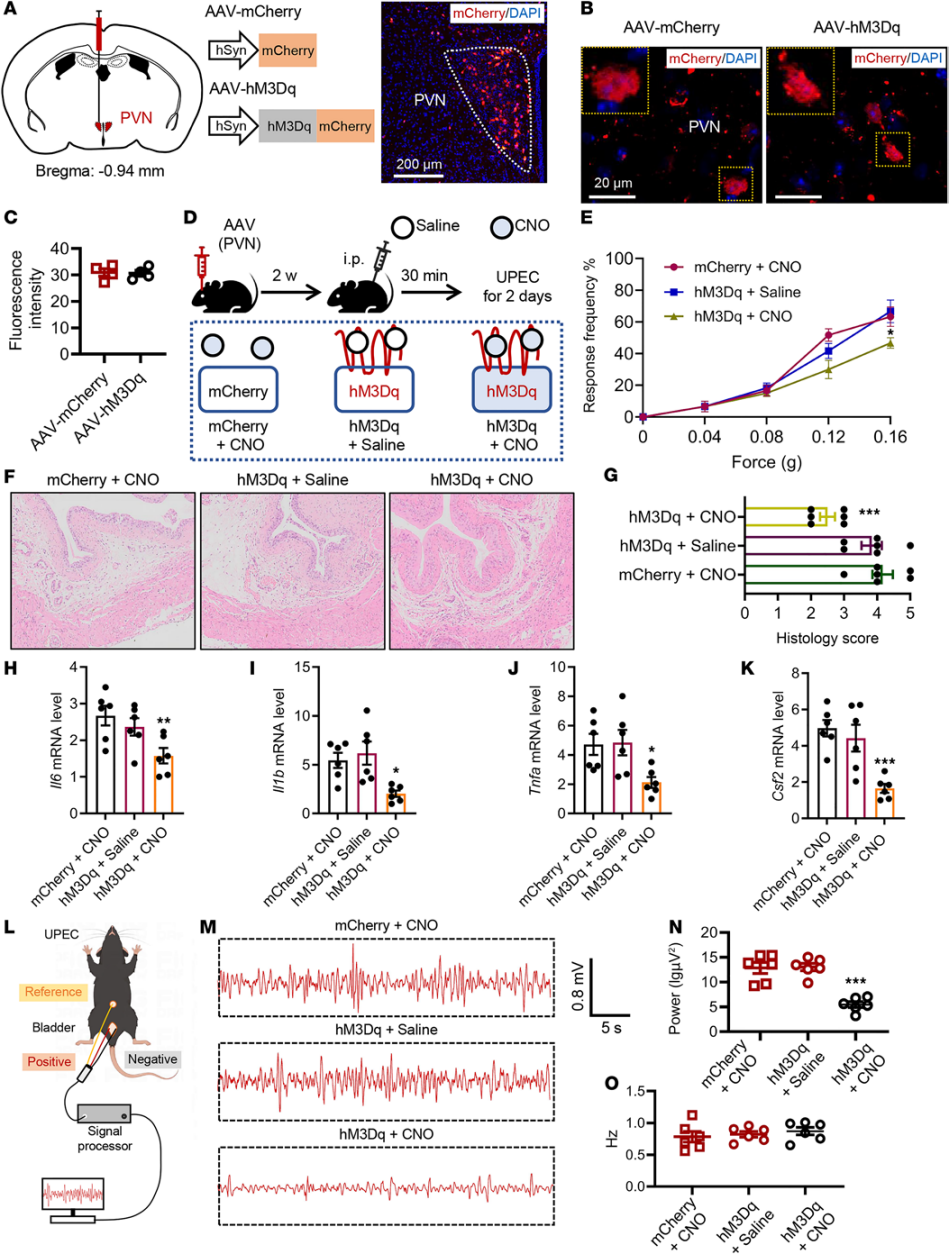
Chemogenetic activation of PVN neurons attenuates bladder inflammation during UPEC infection. (**A**) Left: Diagram of the injection of AAV-mCherry or AAV-hM3Dq virus into the PVN. Right: representative images of virus expression in the PVN on day 14. Scale bar, 200 μm. (**B**) The AAV-mCherry or AAV-hM3Dq virus acts on PVN neurons. Scale bar, 20 μm. (**C**) Fluorescence intensity of mCherry in PVN (*n* = 4 mice per group). (**D**) Diagram of CNO (5 mg/kg) intraperitoneal injection procedure activating PVN neurons in UPEC mice. (**E**) Mice were assessed for pelvic tactile allodynia/hyperalgesia (*n* = 10 mice per group). *P < 0.05 vs. mCherry + CNO group. (**F**) H&E staining of bladders. (**G**) Histology scores were assessed after infection (*n* = 6 mice per group). ***P < 0.001 vs. mCherry + CNO group. (**H**–**K**) The mRNA levels of *Il6*, *Il1b*, *Tnfa*, and *Csf2* of mice (*n* = 6 mice per group). *P < 0.05, **P < 0.01 vs. mCherry + CNO group. (**L**) Diagram illustrating the localization of LFP electrode implants into the bladder wall. (**M**) Representative examples of LFP traces from the bladder wall. (**N**) LFP power in the bladder wall within 1 minute (*n* = 6 mice per group). ***P < 0.001 vs. mCherry + CNO group. (**O**) LFP frequency in the bladder wall within 1 minute (*n* = 6 mice per group). ***P < 0.001 vs. mCherry + CNO group. Results are presented as mean ± SEM and analyzed by 2-tailed Student’s *t* tests (**E**) or 1-way ANOVA with Tukey’s corrections for multiple comparisons (**G**–**K** and **N**).

In previous research, we found UTIs associated with PVN neurons. Here, we want to know whether regulating PVN alleviates UTI-induced bladder inflammation. A chemogenetic technique was employed with AAV-hM3Dq injection into the PVN region (Figure 4A). The AAV-hM3Dq and the control AAV-mCherry virus-infected neurons were both visible in the PVN region, with no observed expression differences (Figure 4, B and C). hM3Dq (Gq) served as a designer receptor exclusively activated by designer drug that only responded to clozapine *N*-oxide (CNO). We intraperitoneally injected CNO (5 mg/kg) into mice to activate the PVN neuronal expression of hM3Dq bilaterally. Three groups of mice were injected with CNO or saline 30 minutes before UPEC treatment (Figure 4D).

In the context of concurrent UPEC infection, a substantial reversal of pain behavior was observed in mice injected with the hM3Dq-carrying virus and treated with CNO (PVN neurons activated) (Figure 4E). Bladder histology was examined using H&E staining, revealing fewer lesions and reduced inflammatory infiltration in the hM3Dq + CNO group (Figure 4, F and G). Inflammatory factors were measured in the bladder samples obtained from the 3 previously mentioned groups of mice. The hM3Dq + CNO mice showed a substantial decrease in *Il1b*, *Il6*, *Tnfa*, and *Csf2* compared with the other groups (Figure 4, H–K). Furthermore, we assessed the LFP of bladder nerve fibers (Figure 4L), revealing a reversal of abnormal discharge in mice following hM3Dq activation in the PVN (Figure 4, M–O). In brief, the data indicate that PVN activity is involved in bladder inflammation and that its activation could attenuate UTIs.

### Psychological stress affects bladder inflammation through glucocorticoids and HPA axis.

**Figure 5:**
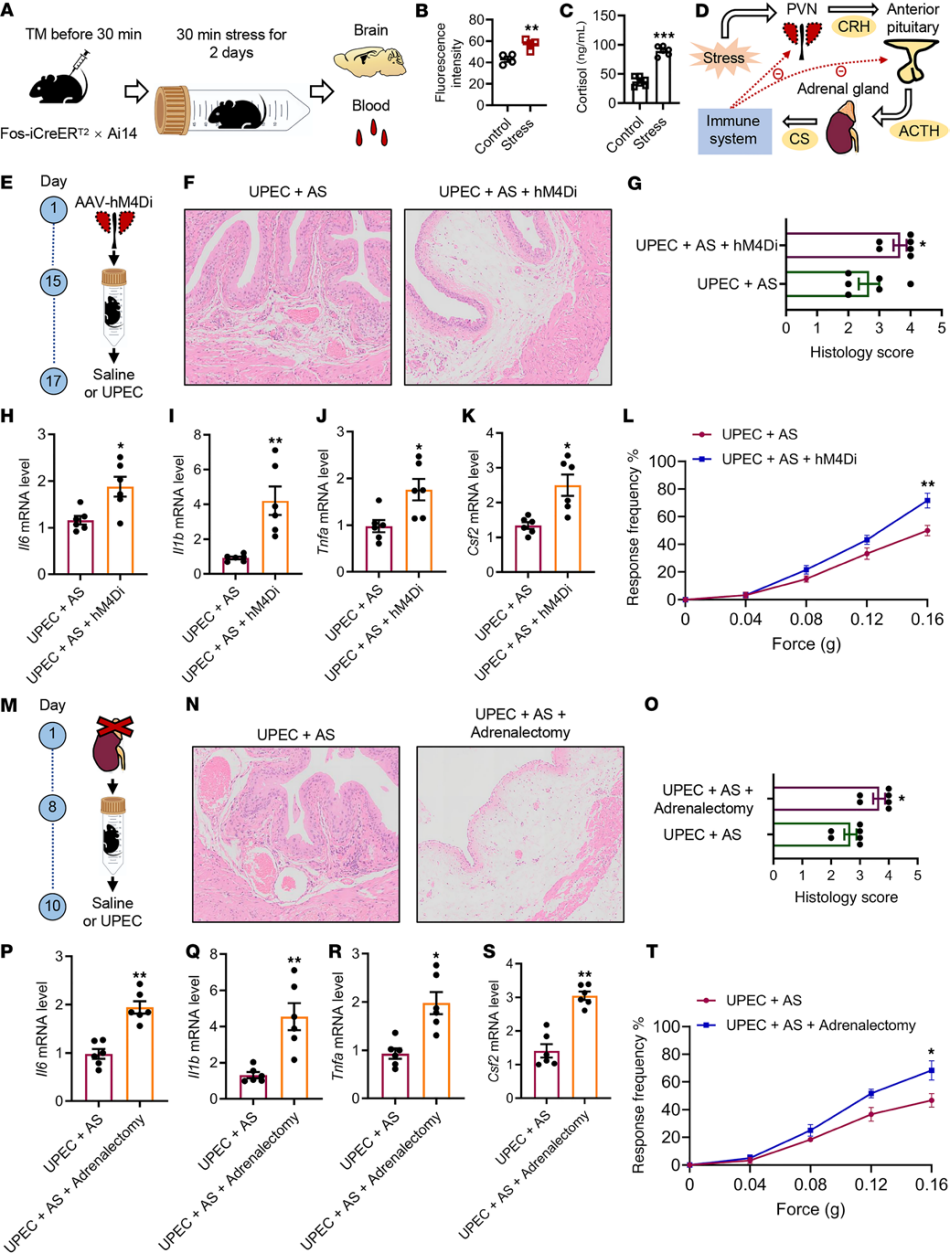
AS alleviates bladder inflammation via glucocorticoids and HPA axis during UPEC infection. (**A**) Experimental flowchart. After 30-minute stress for 2 days, brain tissue and blood samples were collected. (**B**) Fluorescence intensity of Fos^+^ in PVN (*n* = 4 mice per group). **P < 0.01 vs. control group. (**C**) Blood serum glucocorticoid levels (*n* = 6 mice per group). ***P < 0.001 vs. control group. (**D**) The flowchart shows that the HPA axis mediates the feedback between stress and the immune system. (**E**) Experimental flowchart. The PVN brain area was inhibited and the HPA axis was blocked. (**F**) H&E staining of bladders from AS-UPEC infection mice and AS-UPEC infection mice with PVN-hM4Di. (**G**) Histology scores (*n* = 6 mice per group). *P < 0.05 vs. UPEC + AS group. (**H**–**K**) The mRNA levels of *Il6*, *Il1b*, *Tnfa*, and *Csf2* of mice (*n* = 6 mice per group). *P < 0.05, **P < 0.01 vs. UPEC + AS group. (**L**) Mice were assessed for pelvic tactile allodynia/hyperalgesia (*n* = 10 mice per group). **P < 0.01 vs. UPEC + AS group. (**M**) Experimental flowchart. Mouse adrenalectomy and the HPA axis was blocked. (**N**) H&E staining of bladders from AS-UPEC infection mice and AS-UPEC infection mice with adrenalectomy. (**O**) Histology scores (*n* = 6 mice per group). *P < 0.05 vs. UPEC + AS group. (**P**–**S**) The mRNA levels of *Il6*, *Il1b*, *Tnfa*, and *Csf2* of mice (*n* = 6 mice per group). *P < 0.05, **P < 0.01 vs. UPEC + AS group. (**T**) Mice were assessed for pelvic tactile allodynia/hyperalgesia (*n* = 10 mice per group). *P < 0.05 vs. UPEC + AS group. All results are presented as mean ± SEM and analyzed by 2-tailed Student’s *t* tests.

In mice, we verified that activated PVN alleviates UTI-induced bladder inflammation. We next asked how psychological stress is transmitted from the brain to the bladder. After 30 minutes of stress for 2 days, we collected brain tissue and blood samples from Fos-iCreER^T2^ Ai14 mice (Figure 5A). Interestingly, the activated PVN neuron (Figure 5B) and glucocorticoid (Figure 5C) levels were substantially increased in mice after acute restraint stress. PVN is a key brain region that regulates the HPA stress axis response. Upon exposure to a stressor, neurosecretory cells in the PVN release CRH, which stimulates the anterior pituitary to release adrenocorticotropic hormone (ACTH). ACTH then stimulates the release of glucocorticoids from the adrenal gland. Similarly, given the intricate and dynamic nature of the body, the immune system is capable of providing negative feedback to the hypothalamus and pituitary gland (Figure 5D).

To evaluate the influence of glucocorticoid release on UTIs, our subsequent objective was to elucidate the mechanisms by which the brain regulates bladder inflammation. First, we inhibited PVN neurons to block the brain-mediated induction of CRH release (Figure 5E). The effect of AS on UPEC-induced inflammation of bladder was efficiently blocked. H&E staining of the bladder revealed that inhibition of PVN neurons resulted in mucosal edema and infiltration of inflammatory cells (Figure 5, F and G). Additionally, PVN neuron inhibition led to upregulation of *Il1b*, *Il6*, *Tnfa*, and *Csf2* (Figure 5, H–K) in bladders. Compared with the control group, mice with inhibited PVN neurons exhibited more pronounced pain behavior (Figure 5L). Second, we performed adrenalectomy to block the brain-mediated induction of glucocorticoid release (Figure 5M). Similarly, glucocorticoid depletion by adrenalectomy blocked the stress-induced inflammation mitigation of the bladder, as documented by histology (Figure 5, N and O), pro-inflammatory cytokines (Figure 5, P–S), and pain behavior (Figure 5T), suggesting that glucocorticoids mediated the beneficial effect of stress on bladder inflammation. Together, these data suggest that the HPA axis counteracts inflammation during AS.

### Reactivation of PVN neuronal ensembles captured during AS downregulates the inflammatory response.

**Figure 6:**
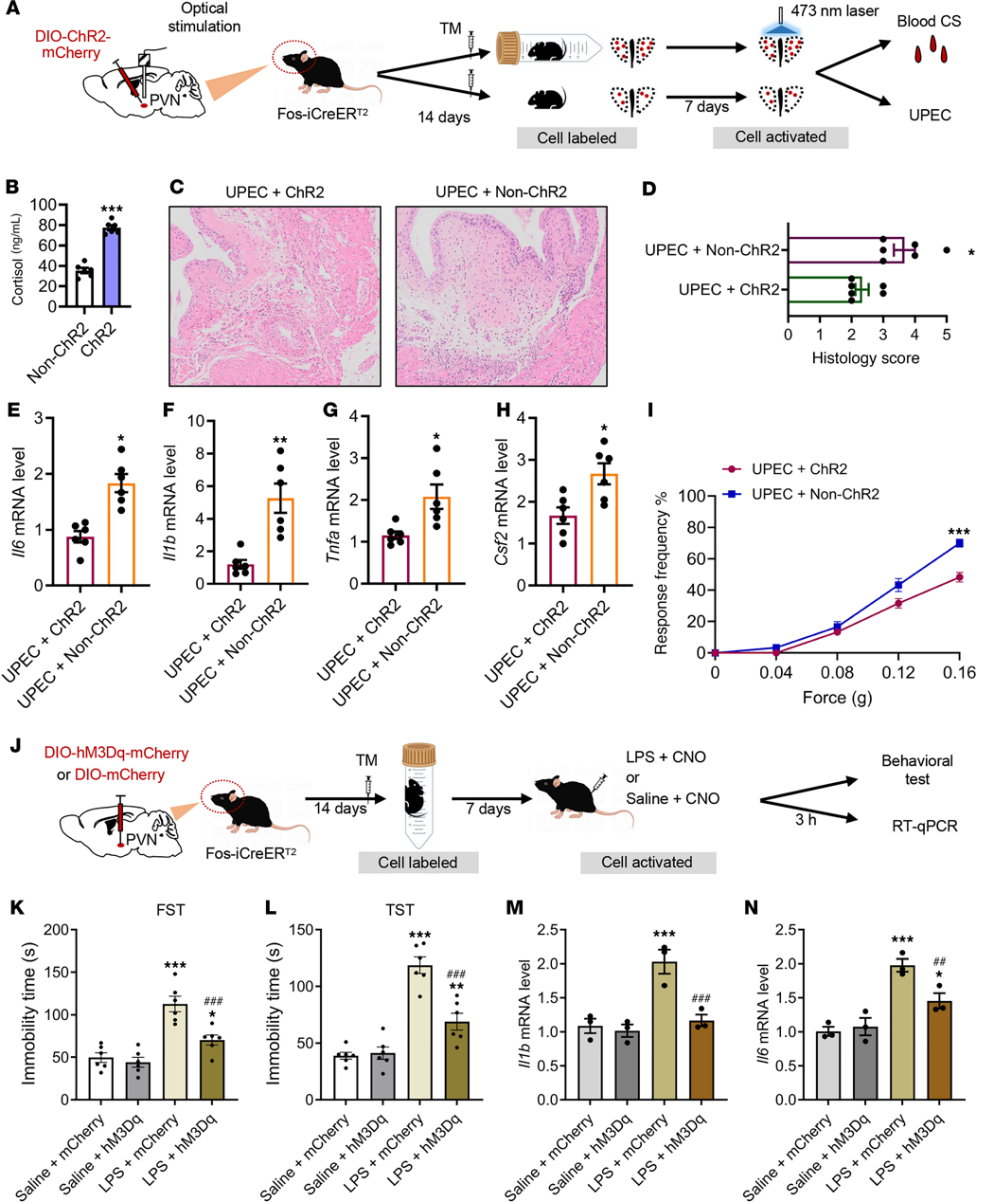
Reactivation of AS-encoded PVN neuronal ensembles suppresses bladder inflammation and depression- and anxiety-like behaviors. (**A**) Experimental flowchart. Optogenetically labeled (during stress) and activated PVN cells in Fos-iCreER^T2^ mice (control or UPEC treated). (**B**) Blood serum glucocorticoid levels (*n* = 6 mice per group). ***P < 0.001 vs. non-ChR2 group. (**C**) H&E staining of bladders from AS-UPEC infection mice with ChR2 and UPEC infection mice with non-ChR2. (**D**) Histology scores were assessed after infection (*n* = 6 mice per group). *P < 0.05 vs. UPEC + ChR2 group. (**E**–**H**) The mRNA levels of *Il6*, *Il1b*, *Tnfa*, and *Csf2* of mice (*n* = 6 mice per group). *P < 0.05, **P < 0.01 vs. UPEC + ChR2 group. (**I**) Mice were assessed for pelvic tactile allodynia/hyperalgesia with von Frey filaments before (baseline) and after infection (*n* = 10 mice per group). ***P < 0.001 vs. UPEC + ChR2 group. (**J**) Experimental flowchart. Chemogenetically labeled (during stress) and activated PVN cells in Fos-iCreER^T2^ mice (saline or LPS treated). (**K**) FST results show mouse immobility time (*n* = 6 mice per group). *P < 0.05, ***P < 0.001 vs. saline + mCherry group. ^###^*P* < 0.001 vs. LPS + mCherry group. (**L**) TST results show mouse immobility time (*n* = 6 mice per group). **P < 0.01, ***P < 0.001 vs. saline + mCherry group. ^###^*P* < 0.001 vs. LPS + mCherry group. (**M** and **N**) The mRNA levels of *Il1b* and *Il6* of mice (*n* = 3 mice per group). ***P < 0.001 vs. saline + mCherry group. ^##^*P* < 0.01, ^###^*P* < 0.001 vs. LPS + mCherry group. Results are presented as mean ± SEM and analyzed by 2-tailed Student’s *t* tests (**B** and **D**–**I**) or 1-way ANOVA with Tukey’s corrections for multiple comparisons (**K**–**N**).

To further clarify this question, we labeled PVN neurons during AS and free activity in mice, respectively. We optogenetically activated the *TRAP*ed neurons and evaluated their effects on the immune response (Figure 6A). The results showed that reactivated AS period PVN neurons substantially increased glucocorticoid level in blood compared with the non–channelrhodopsin-2 (non-ChR2) group (Figure 6B). Remarkably, activating the PVN *TRAP*ed neurons during AS (but not during free activity) substantially reduced bladder inflammation induced by UPEC infection. We initially investigated the impact of PVN *TRAP*ed activation on inflammation and demyelination through histological analysis (Figure 6C). The histology scores showed an increase in the non-ChR2 group (Figure 6D). Additionally, we observed that activating the PVN *TRAP*ed neurons reversed the mRNA levels of *Il1b*, *Il6*, *Tnfa*, and *Csf2* (Figure 6, E–H) in the bladder of the ChR2 group. Ultimately, activating the PVN *TRAP*ed neurons substantially ameliorated pain behavior severity (Figure 6I).

To examine the nature of the immune information encoded by neuronal ensembles in the PVN, we added a different immune challenge, lipopolysaccharide (LPS). This well-defined inflammatory process affects the body systemically, and it is immunologically distinct from UPEC-induced UTIs. As in the previous experiment, we injected TM to label the active PVN neurons during the AS treatment. After 7 days of recovery, we reactivated the captured PVN neuronal ensembles (by CNO injection) and intraperitoneally injected LPS or saline in mice (Figure 6J). Depression-like behavior was an indicator of systemic inflammation. Consistent with our hypothesis, the immobility time was substantial increased in the LPS treatment group, while the mice with activated PVN *TRAP*ed neurons ameliorated depression-like behaviors (Figure 6, K and L). In addition, reactivation of PVN neurons during AS substantially reduced the levels of inflammatory cytokines *Il1b* and *Il6* compared with the LPS treatment group (Figure 6, M and N).

In conclusion, these data demonstrate that AS-activated PVN neurons may play a crucial role in antiinflammatory reflexes by the brain-body circuit.

### Glucocorticoid signal controls ILC2.

**Figure 7:**
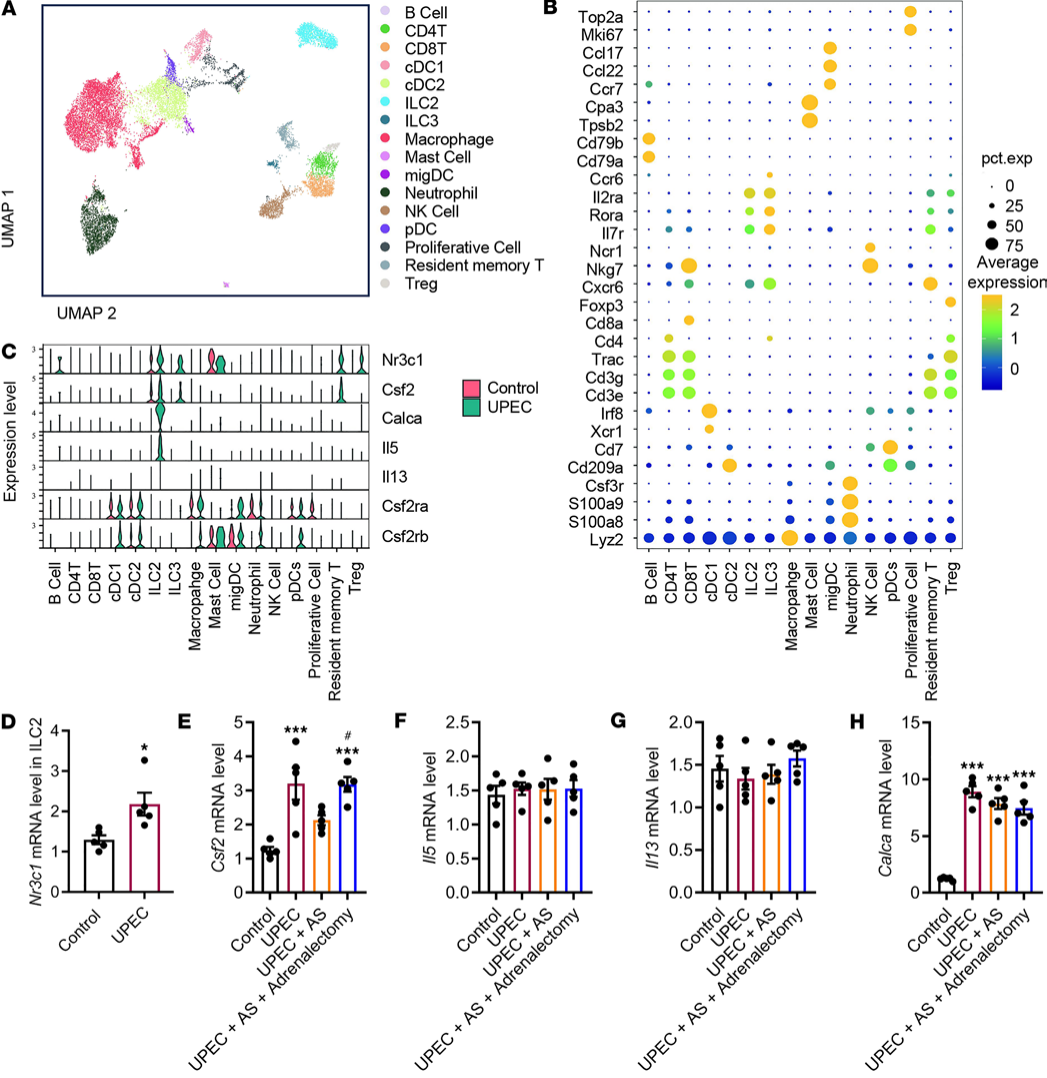
Glucocorticoid signaling inhibits Csf2 secretion by ILC2 cells during UPEC infection. (**A**) Uniform manifold approximation and projection (UMAP) plot illustrating the integrated analysis of immune cells isolated from mouse bladders treated with UPEC or untreated. cDC, conventional dendritic cell; migDC, migratory dendritic cell; pDC, plasmacytoid dendritic cell. (**B**) Dot plot showing the expression of immune cell markers identified in each population. (**C**) Violin plots highlighting Nr3c1, Csf2, Calca, Il5, Il13, Csf2ra, and Csf2rb derived from the overlaid scRNA-Seq data of UPEC or untreated mice. (**D**) RT-qPCR of the GR using RNA isolated from bladder ILC2 (control or UPEC treated) (*n* = 5 mice per group). *P < 0.05 vs. control group. (**E**–**H**) RT-qPCR of *Csf2*, *Il5*, *Il13*, and *Calca* using RNA isolated from bladder ILC2 (*n* = 5 mice per group). *P < 0.05, ***P < 0.001 vs. control group. ^#^*P* < 0.05 vs. UPEC + AS. Results are presented as mean ± SEM and analyzed by 2-tailed Student’s *t* tests (**D**) or 1-way ANOVA with Holm-Šidák corrections for multiple comparisons (**E**–**H**).

Because activation of the HPA stress axis suppressed bladder inflammation, we next sought to identify the cell populations within the bladder immune microenvironment that respond to glucocorticoids. We utilized publicly accessible single-cell sequencing databases (16) to comprehensively characterize the immune cell population in the bladder. Subsequently, single-cell RNA-Seq (scRNA-Seq) profiles were generated for 16,844 immune cells obtained from both naive mice and mice following UPEC infection. The uniform manifold approximation and projection (UMAP) algorithm identified 16 distinct cell clusters (Figure 7A). Each cell cluster was annotated based on the unique expression of characteristic marker genes, and representative genes were visualized as a dot plot (Figure 7B). Several recent studies have demonstrated the essential role of macrophages in the induction of inflammation. Interestingly, our scRNA-Seq analysis revealed that glucocorticoid receptor (Nr3c1, hereafter GR) mRNA was not detected within the macrophage cluster. Instead, expression of GR was found to be restricted to ILC2, ILC3, and mast cells and resident memory T cells and Tregs (Figure 7C). In a prior investigation, we discerned the participation of bladder ILC2 in the exacerbation of UTIs. We then inquired whether glucocorticoids have the ability to inhibit the transcription of genes encoding pro-inflammatory cytokines within ILC2 cells. Analysis of scRNA-Seq revealed a high abundance of Csf2 within ILC2 cells, which are well-known direct targets of glucocorticoids (26). Furthermore, it was observed that the receptors for Csf2 are widely expressed by bladder leukocytes (Figure 7C). We isolated flow-sorted ILC2 cells from mouse bladder to monitor GR mRNA level dynamics and subsequently evaluated glucocorticoid-mediated suppression of pro-inflammatory gene expression through Csf2 transcriptional profiling. Real-time quantitative PCR (RT-qPCR) analysis of bladder ILC2 revealed a marked upregulation of GR mRNA in the UPEC group (Figure 7D). The secretion of *Csf2* by ILC2 was substantially reduced in mice experiencing AS and subsequently elevated following adrenalectomy (Figure 7E). However, no differences were present in the expression of *Il5*, *Il13*, and *Calca* between the 2 groups (Figure 7, F–H).

### Genetically deleting Csf2 in ILC2 cells decreases inflammation.

**Figure 8:**
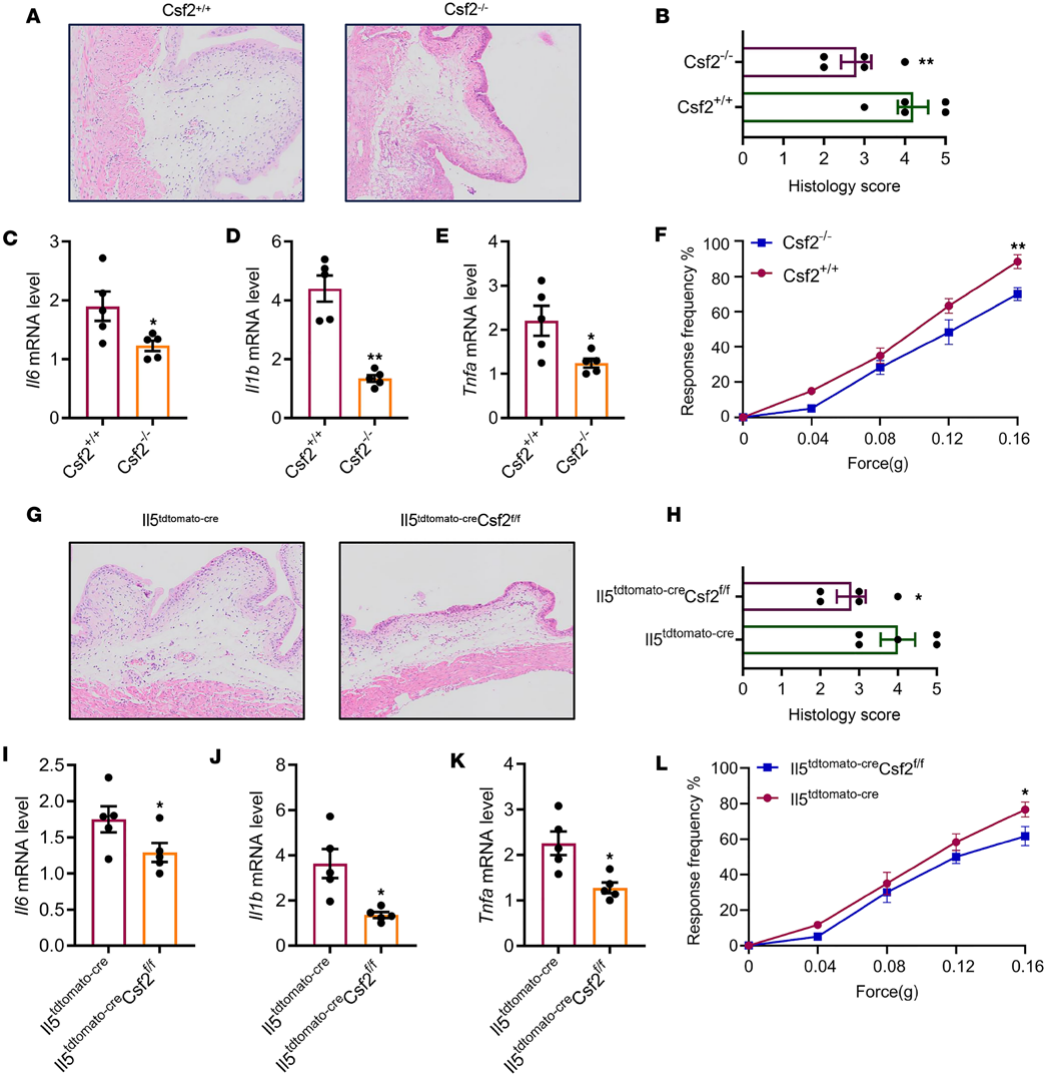
ILC2 cells exacerbate inflammation and voiding behavior through Csf2 secretion during UPEC infection. (**A**) H&E staining of bladders from Csf2^+/+^ mice and Csf2^–/–^ mice with UPEC infection. (**B**) Histology scores (*n* = 5 mice per group). **P < 0.01 vs. Csf2^–/–^ group. (**C**–**E**) The mRNA levels of *Il6*, *Il1b*, and *Tnfa* of mice (*n* = 5 mice per group). *P < 0.05, **P < 0.01 vs. Csf2^–/–^ group. (**F**) Mice were assessed for pelvic tactile allodynia/hyperalgesia (*n* = 10 mice per group). **P < 0.01 vs. Csf2^–/–^ group. (**G**) H&E staining of bladders from Il5^tdtomato-cre^ mice and Il5^tdtomato-cre^ Csf2^fl/fl^ mice with UPEC infection. (**H**) Histology scores (*n* = 5 mice per group). *P < 0.05 vs. Il5^tdtomato-cre^ Csf2^fl/fl^ group. (**I**–**K**) The mRNA levels of *Il6*, *Il1b*, and *Tnfa* of mice (*n* = 5 mice per group). *P < 0.05 vs. Il5^tdtomato-cre^ Csf2^fl/fl^ group. (**L**) Mice were assessed for pelvic tactile allodynia/hyperalgesia (*n* = 10 mice per group). *P < 0.05 vs. Il5^tdtomato-cre^ Csf2^fl/fl^ group. All results are presented as mean ± SEM and analyzed by 2-tailed Student’s *t* tests.

Csf2 was initially characterized as a cytokine that facilitates the differentiation of granulocytes and macrophages from bone marrow precursors (27) and plays a pivotal role in UPEC-induced acute pyelonephritis (28). We utilized Csf2^–/–^ mice to investigate the impact of Csf2 deficiency on bladder inflammation in UPEC-infected mice. We observed that Csf2^–/–^ mice exhibited milder histopathological scores (Figure 8, A and B), lower expression of pro-inflammatory cytokines (Figure 8, C–E), and decreased pelvic allodynia (Figure 8F) compared with WT littermates. Next, we tried to investigate the function of Csf2-producing ILC2 cells during UPEC infection. Il5^tdtomato-cre^ mice have been widely used to target ILC2 (29). Depletion of ILC2 resulted in decreased inflammation and altered voiding behavior in the context of UPEC infection (16). We employed Csf2^fl/fl^ mice in conjunction with Il5^tdtomato-cre^ mice to achieve genetic ablation of Csf2 in ILC2 cells. Il5^tdtomato-cre^ Csf2^fl/fl^ mice exhibited reduced histopathological scores (Figure 8, G and H), expression of pro-inflammatory cytokines (Figure 8, I–K), and pelvic allodynia compared with Il5^tdtomato-cre^ mice (Figure 8L).

## Discussion

A number of epidemiological studies have affirmed that psychological stress and UTIs interact and have a causal relationship with each other (30, 31), but the mechanistic pathways remained poorly understood and required further investigation. This study demonstrated that acute restraint stress protected the bladder from UTIs, and PVN activation was necessary and sufficient for this effect to occur. Restraint stress and the PVN regulate the HPA axis response to produce an equivalent degree of protection against UTIs by 3 measures: bladder pain score, bladder damage histological analysis, and inflammatory factor levels. In addition, we demonstrate that the reactivation of PVN AS-*TRAP*ed neurons is sufficient to induce a vigorous antiinflammatory reflex and ameliorate UTIs consequently. Finally, we observed an elevation in glucocorticoids following HPA axis activation, along with a high expression of Nr3c1 in ILC2 cells. These are crucial for the regulation of bladder inflammation and pelvic allodynia during UPEC infection.

It is important to note that UTIs are often associated with inflammation (32) and neuronal hypersensitivity (33). The emerging field of neuroimmunology investigates the interactions between neurons, leukocytes, and their signaling molecules in both normal and pathological conditions (34, 35). Although the central regulation of autonomic functions like respiration and digestion has been extensively researched, the specific neuronal populations governing the top-down brain-body circuit that modulates systemic immune responses remain less explored (36). It is widely reported that stress (8) and pain (37) play critical roles in immune regulation and inflammatory processes throughout the body. Stress can alter the immune experience encoded by the brain (17). Yet, previous studies suggest that acute stressors stimulate the immune system to enhance the clearance of pathogens, whereas CS exerts immunosuppressive effects. These differences may derive from divergences in monocyte mobilization dynamics in CS (8, 38, 39). In line with our findings, we found that AS treatment inhibited bladder inflammation in UTI mice, while CS increased bladder infections. However, the mechanism underlying this difference requires further investigation. PVN, as a critical region in the hypothalamus, plays a key role for stress signals to induce the glucocorticoid response (40). Prior research has indicated that in mammals, the HPA axis serves as the primary neuroendocrine system activated in response to stressors, leading to increased levels of glucocorticoids in the periphery (41). Our results innovatively indicate that the PVN mediates AS-induced antiinflammatory reflex in mice with UTIs.

In addition, numerous recent studies demonstrate that brain neurons can acquire and retrieve specific event-related information (42, 43). In the field of neuroscience, this type of information encoding is reminiscent of memory traces (44). An extension of the c-Fos–labeling approach is *TRAP* (17, 43). In the presence of TM, active cells expressing c-Fos are permanently tagged with a fluorescent protein, enabling visualization of neurons active at a specific time point in *TRAP* mice. Recent studies have primarily demonstrated such memory traces through the retrieval of fear-related behaviors, facilitated by the reactivation of hippocampal neuronal ensembles captured during fear conditioning (45, 46). Similarly, in our paradigm, using *TRAP* mice showed that reactivation of PVN neurons captured under a unique AS condition was sufficient to induce a related antiinflammatory response. However, these principles may also apply to the extensively explored neural region comprising the LH, mPFC, ARH, and hippocampus in the context of inflammation and may be further extended to other brain areas involved in memory ensembles in future studies.

Glucocorticoids primarily signal by binding to the GR, and our findings demonstrate that the GR is widely expressed in various immune cells including ILC2, consistent with their established role in regulating inflammation. ILC2 cells play a crucial role in shaping immunity during homeostasis and pathogen insult (47, 48). Due to the absence of antigen-specific receptors, ILC2 cells are localized within mucosal interfaces, allowing for a prompt and vigorous cytokine reaction (47, 49). It is important to note that UTIs are often associated with inflammation (50) and neuronal hypersensitivity (51). Our previous findings demonstrated that bladder ILC2 cells contribute to the exacerbation of bladder inflammation and pelvic allodynia during UPEC infection, revealing the functional complexity of ILC2 cells in UTIs (16). In this study, we demonstrate that bladder ILC2 cells express Csf2 that was known to modulate macrophage activity (52, 53) and chronic prostatitis/chronic pelvic pain syndrome (54). The expression of Csf2 secreted by ILC2 cells was substantially suppressed following adrenalectomy. In the current report, we present evidence demonstrating that Csf2^–/–^ mice and Il5^tdtomato-cre^ Csf2^fl/fl^ mice exhibited reduced severity of inflammation in comparison with control mice in UTIs. This suggests that dysregulation of Csf2 may drive the primary activation of inflammation and serve as the initiating factor for bladder immunity. Our findings suggest that a nonantibiotic management strategy (CNS regulates the brain-body circuit) could mitigate UTI inflammation by regulating bladder ILC2, potentially offering an effective treatment approach in the future. There are still several limitations present. First, we have not fully excluded the possibility that glucocorticoids may directly suppress inflammation through other immune cells during bladder infection. Second, in addition to ILC2, it has been observed that LPS could stimulate the production of Csf2 from resident tissue cells, including urothelial cells (55). Furthermore, the effects of male mice were not explored in our study. In summary, we show that AS activates the PVN to promote glucocorticoid release, then inhibits the pro-inflammatory cytokine Csf2 expression by ILC2 cells. Our findings reveal the potential of the brain-body circuit as a means of suppressing UTI inflammation. Thus, this study adds another perspective to the understanding of these pathological conditions and, presumably, an avenue for therapeutic intervention.

## Methods

### Sex as a biological variable.

As cystitis is more prevalent in female patients, except for the experiments to mark excitatory brain areas (TM is susceptible to estrogen level interference), our experiments were all performed using female mice.

### Animals.

The 8- to 10-week-old female C57BL/6J mice were acquired from Beijing Vital River Laboratory Animal Technology Co. Ltd. The 8- to 10-week-old male and female Fos-iCre-ER^T2^ mice, Gt (ROSA)-Ai14-tdTomato mice, and Il5^tdtomato-cre^ mice were obtained from Jackson Laboratory. Csf2^–/–^ mice and Csf2^fl/fl^ mice were obtained from Shanghai Model Organisms Center, Inc. The mice were maintained in a light/dark cycle (12 hours of light and 12 hours of dark), light on from 8:00 am to 8:00 pm, and in a temperature-constant environment (22.5°C). The mice had ad libitum access to food and water and were housed in polycarbonate cages.

### Murine UTI model.

UPEC strain CFT073 was purchased from ATCC. Mice were anesthetized, and cystitis was induced by transurethral inoculation of 30 μL of a UPEC suspension in PBS (1× 10^7^ to 2 × 10^7^ CFU) via a catheter designed for mice (inner diameter 0.28 mm). The control group received saline treatment via catheter.

### Psychological stress.

For acute restraint stress, mice were placed into 50 mL polypropylene conical tubes with ventilation caps for 30 minutes for 2 days. For chronic restraint stress, mice were placed into 50 mL polypropylene conical tubes with ventilation caps for 4 hours for 14 days.

### Drugs, reagents, and virus.

CNO was purchased from AbMole BioScience Co., Ltd. TM and LPS were purchased from MilliporeSigma. PRV were purchased from Brain Case Co., Ltd. AAV-hSyn-mCherry and AAV-hSyn-hM3D (Gq)-mCherry viruses were purchased from Genechem Co., Ltd. (Shanghai, China). pAAV-CMV-GCaMP6s-P2A-NLS viruses were purchased from SunBio Biomedical Technology Co., Ltd. (Shanghai, China). All viruses were stored at −80°C until use, and the viral titers were more than 10^12^ viral particles/mL.

### Stereotaxic surgery and virus injection.

The procedure for virus injection and stereotaxic surgery was adopted from that as described in a previous study (56). Briefly, the mice were deeply anesthetized with sodium pentobarbital (57-33-0, MilliporeSigma) (50 mg/kg) and then positioned on a stereotaxic instrument (Model 900, Kopf). Viruses were bilaterally injected into the PVN regions (anterior-posterior [AP], −0.94 mm from bregma; medial-lateral [ML], ± 0.20 mm; dorsal-ventral [DV], −4.70 mm) at a flow rate of 0.04 μL/min. The pipette was slowly withdrawn at least 10 minutes after the injection to prevent backflow of the virus upon retraction. The incision was closed with sutures, and the mice were allowed to recover in their original cages and housed for 2 weeks before further experiments.

### HE staining.

The mouse bladder tissues were kept in 10% formalin and embedded in paraffin. Paraffin sections were stained for hematoxylin and eosin. Histological scoring was based on parameters like edema, bleeding, inflammatory cell infiltration, and epithelial changes, with scores ranging from 0 to 5 (57). 0: Morphologically unremarkable, showing minimal or no inflammation or epithelial alterations. 1: Minimal inflammatory infiltrate with occasional neutrophils or lymphocytes in the lamina propria. 2: Minimal to mild inflammatory infiltrate in the lamina propria with scattered neutrophils or lymphocytes. 3: Mild to moderate inflammatory infiltrate in the lamina propria, extending into the muscularis propria. 4: Moderate inflammation with frequent neutrophils and lymphocytes in both layers. 5: Severe inflammation in both layers with urothelial ulceration, severe edema, hemorrhage, and fibrin deposition.

### Immunofluorescence assays.

Mice were deeply anesthetized with sodium pentobarbital (50 mg/kg). The brains were removed and frozen coronal sections were cut at a thickness of 30 μm using a freezing microtome. The bladder sections were incubated overnight at 4°C with primary anti-Uroplakin3a (A10034, Abclonal). After washing with PBS, the slides were incubated with secondary Alexa Fluor 488 (ab150077, Abcam) for 1 hour. After washing with PBS, we stained the slides with DAPI (28718-90-3, MilliporeSigma) for 10 minutes. We obtained images using the LSM880 confocal microscope (ZEISS) and Olympus confocal microscope, then analyzed using ImageJ version 1.8.0 software (NIH).

### Peripheral nociception testing.

Mice were tested at 2 days after infection. Referred hyperalgesia and tactile allodynia were tested using von Frey filaments applied to the abdomen and plantar region of the hind paw, as previously described (58). Forces of 0.04, 0.08, 0.12, and 0.16 g (Bioseb) were tested for the development of referred hyperalgesia and tactile allodynia. Each filament was applied with a resting period of 5 seconds for a total of 10 times, and repeated stimulation at the same point was avoided to prevent “wind-up” effects or desensitization. Stimulation was confined to the lower abdominal or perineum region. The following conditions were recognized as a positive response to the filament: a) retraction of the abdomen, b) licking and scratching of the pelvic area of stimulation immediately, and c) jumping.

### Fiber photometry recording and analysis.

At 2 weeks after the injection of the AAV-GCaMP6s viruses into the PVN, an optical fiber was implanted 200 μm above the PVN injection site. The custom headpost was affixed to the skull with opaque dental cement, which prevented light leakage from the skull. The fiber photometry recording was conducted shortly after micturition in mice. The RWD Fiber Photometry System utilized 470 nm and 410 nm LEDs for GCaMP6s (calcium) and control emission fluorescence, respectively. Emitted signals were captured at 120 Hz with alternating pulses of 470 nm and 410 nm light, resulting in frame rates of 60 Hz for GCaMP6s and control signals. Calcium activity data and baseline-corrected calcium signals were analyzed using the RWD ORFS Data Process software. We derived the value of the GCaMP6s signal F during the designated epochs as F470/F410, calculating ΔF/F = (F – F0)/F0, where calcium signals greater than 3 SDs were considered events. The average of the peak ΔF/F values and the number of events per minute for each mouse were analyzed. The AUC baseline was set as a 5-second period before the onset of immobility (onset) and a 5-second period after the end of immobility (end).

### LFP.

The LFP recording was conducted shortly after micturition in mice. Mice were stably anesthetized and implanted with a single tungsten wire to record LFP in the PVN regions (AP, − 0.94 mm from bregma; ML, ± 0.20 mm; DV, − 4.70 mm). The reference wire was in the symmetrical position from the single tungsten wire, and the ground wire was set on the cerebellum using miniature stainless-steel screws and connected the electrodes to the LabChart signal amplifier (AD Instruments). We divided the neuronal oscillations into 5 frequency bands: delta (1~4 Hz), theta (4~8 Hz), alpha (8~13 Hz), beta (13~30 Hz), and gamma (30~100 Hz). Data were recorded and analyzed with the use of the LabChart software.

We used LFP experiments to detect bladder nerve fiber activity in mice. The mice were anesthetized shortly after micturition. The electrodes were positioned as follows: the positive electrode was placed in the target recording area, specifically the bladder nerve fibers. The negative electrode was positioned on the bladder wall, opposite the horizontal line of the positive electrode. The reference electrode was placed subcutaneously in the abdominal cavity. Data were recorded and analyzed with the use of the LabChart software.

### RT-qPCR analysis.

Total RNA was extracted by using TRIzol (Invitrogen) according to the manufacturer’s instructions. Extracted RNA was synthesized into cDNA using PrimeScript RT Master Mix (Accurate Biology). Synthesized cDNA was used for RT-qPCR with SYBR Green Pro Taq Mix (Accurate Biology) following the manufacturer’s instructions. The result of mRNA relative expression levels was analyzed by the 2^−ΔΔCt^ method. *GAPDH* was used as the reference gene. The primers used in this study are listed in Supplemental Table 1.

### Adrenalectomy.

In brief, a midline incision was made, and the abdominal cavity was entered through the abdominal wall, lateral to the dorsal incision. The adrenal gland with the attached fat pad was individually pulled out of the abdominal cavity. The adrenal gland was dissected and the fat pad returned to the abdominal cavity. The process was repeated on the opposite side. The skin incision was closed with wound clips. After surgery, the mice were supplied with 0.9% saline solution as drinking water. Success of the surgical procedure was determined by measuring glucocorticoid levels of randomly selected operation and sham mice.

### Glucocorticoid ELISA.

Serum was separated after blood was collected from the orbital venous plexus of the mice. Blood serum glucocorticoid levels were measured using a commercial mouse Cortisol ELISA kit (glucocorticoids, EM1721, Elabscience Biotechnology Co., Ltd.) according to the manufacturer’s instructions.

### Optogenetics.

The 8- to 10-week-old male Fos-iCre-ER^T2^ mice that received ChR2 in the PVN and optic fiber implantation were allowed to recover for 2 weeks. At 30 minutes before the experiment, TM was injected to label the PVN active neurons in mice with stress or nonstressed mice. Then mice were singly housed and rested for 7 days before the test. Subsequently, blue light (473 nm, 10 ms per pulse, 20 Hz) was used to stimulate labeled PVN neurons for 1 hour. In some mice, serum was separated for a glucocorticoid test following direct blood sampling, while the remaining mice were administered UPEC to assess the ameliorated level of bladder inflammation.

### Behavioral tests.

The behavioral tests related to depression and anxiety were conducted as previously described (56, 59).

### OFT.

The OFT was conducted in a quiet environment using an open-field box (25 × 25 × 45 cm). The bottom of the box was divided into 16 equal squares, with the central area comprising the 4 middle squares. Mice were gently placed in 1 corner of the box and observed for 5 minutes. The mean velocity within the box and the time spent in the central area were recorded and analyzed using the DigBehav video tracking system. The box was wiped with 75% alcohol to dispel the smell after each animal was tested.

### EPM.

The EPM consisted of a central platform with 2 open arms and 2 closed arms arranged at a 90° angle, positioned 40 cm above the floor. The open arms, measuring 30 × 5 cm, had no side or end walls. The closed arms, measuring 30 × 5 cm × 15 cm, were enclosed with side and end walls but open on top. Mice were individually placed on the central platform and given 5 minutes for free exploration. The time spent in the open arms was recorded and analyzed using the TopScan-TopView Behavior Analysis System. After the test, feces were cleaned, and the maze was wiped with 75% alcohol to remove odors.

### FST.

Mice were individually placed in a glass cylinder (12 cm in diameter and 25 cm in height) filled with water for 6 minutes. The depth of water was set to prevent mice’s tails or hind limbs from touching the glass cylinder bottom. Immobility time was defined as animals remaining floating or motionless, only exposing their head above the water surface, and the immobility time was recorded for the last 4 minutes of the 6-minute test period.

### TST.

Mice were individually suspended at 20 cm above the floor with tape placed 15 cm from the distal end of their tail. Immobility time, defined as an absence of body movement, was recorded during the last 4 minutes of the 6-minute test period.

### Flow cytometry.

Mice were humanely euthanized by cervical vertebra dislocation, and bladders were excised. Fresh bladders were rinsed with cold PBS, dissected, minced, and incubated in a solution containing Dispase II (20 mg/mL, MilliporeSigma D4693), DNase I (1 mg/mL, Solarbio D8071), and Liberase TL (0.5 mg/mL, Roche 5401020001) at 37°C for 2 hours. Immune cells were isolated from the digested tissue and filtered through a 100 μm cell strainer. Live and dead cells were distinguished using the Zombie Aqua Fixable Viability Kit (BioLegend 423102). Samples were blocked with 1% anti-mouse CD16/CD32 (Thermo Fisher Scientific 14-0161) in FACS buffer for 15 minutes at 4°C. Cells were stained with antibody cocktails, excluding lineage-positive cells for analysis of ILC2. Lineage marker mix (Lin) contained FITC-Cy7-CD3e, CD5, CD19, B220, Ly6G, CD11b, CD11c, FcεR1, and Ter119; AF700-CD45; APC-CD127 and percp-Klrg1.

### Data availability.

scRNA-Seq datasets specifically focusing on bladder immune cells during UPEC infection have been deposited at NCBI GEO and are publicly available as of the date of publication (GSE259362, GSE260501).

### Statistics.

All data were shown as the means ± SEM. Statistical analyses were performed with GraphPad Prism software (version 9.0). Two-tailed Student’s *t* tests and 1-way or 2-way ANOVA followed by Tukey’s or Holm-Šidák post hoc test were used to perform comparisons between groups. Differences with *P* < 0.05 were considered to indicate statistical significance.

### Study approval.

All animal experiments in this study were approved by the Institutional Animal Care and Use Committee at the Shandong Provincial Hospital (No. 2024-143).

### Data availability.

All data generated or analyzed during this study are included in this published article.

## Author contributions

QR and YL were responsible for conceptualization. DS, KZ, and TD were responsible for methodology. JW and JL were responsible for investigation. QR and QF were responsible for supervision. QR and YL were responsible for writing the initial draft. QR and YL were responsible for reviewing and editing the manuscript.

## Supplementary Material

Supplemental data

Supporting data values

## Version 1 03/18/2025

In-Press Preview

## Version 2 05/08/2025

Electronic publication
