# Supplementary material for: The brain-body circuit mediates acute stress–induced antiinflammatory reflex in bacterial cystitis by suppressing ILC2 activation
Source: JCI Insight. 2025 Mar 18;10(9):e189362. doi: 10.1172/jci.insight.189362 (PMC12128985; doi:10.1172/jci.insight.189362)
Supplement: Supplemental data [file jciinsight-10-189362-s083.pdf]

The brain-body circuit mediates acute stress-induced anti-inflammatory reflex in bacterial cystitis by suppressing ILC2 activation

Authors

Yaxiao Liu[1], Jinhua Wang[2], Junyang Lin[1], Dingqi Sun[1], Kejia Zhu[1], Tongxiang Diao[1], Qiang Fu[1]*, Qingyu Ren[3]*

Affiliations

[1] Department of Urology, Shandong Provincial Hospital Affiliated to Shandong First Medical University, Jinan, Shandong, 250012, P.R. China.

[2] Department of Radiotherapy, Shandong Second Provincial General Hospital, Jinan, Shandong 250000, P.R. China.

[3] Department of Anatomy and Neurobiology, School of Basic Medical Sciences, Shandong University, Jinan, Shandong, 250012, P.R. China.

Correspondence

*Corresponding author.

Qingyu Ren, Email: RQY718374269@163.com.

Qiang Fu, Email: Qiangfu68@163.com

Supplementary Figures

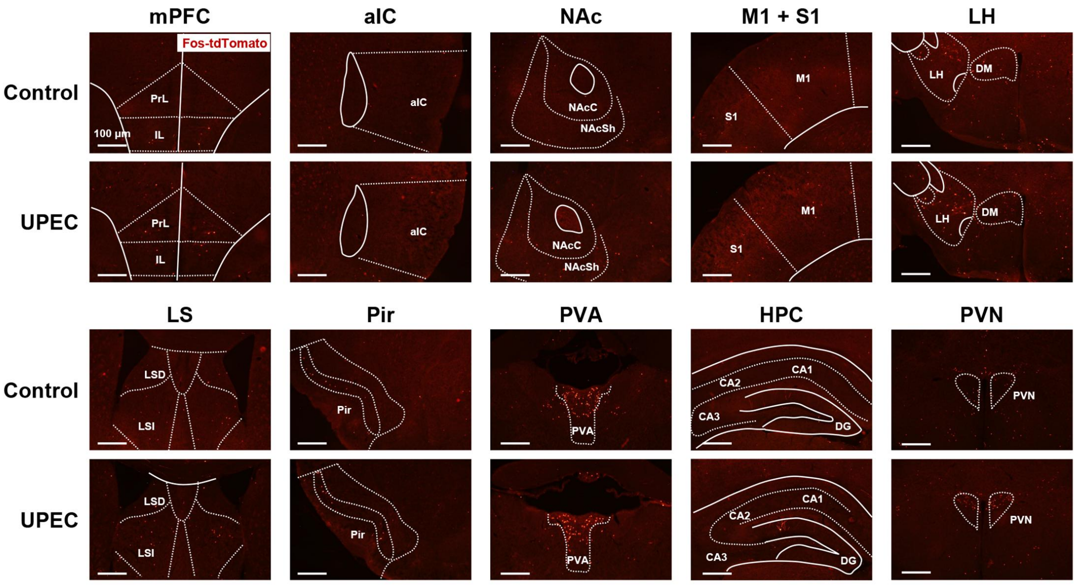

Fig S1. Changes in neuronal activity during UTIs. Representative images of c-Fos expression in several brain neurons labeled with tdTomato (red). Scale bar, 100 μm.

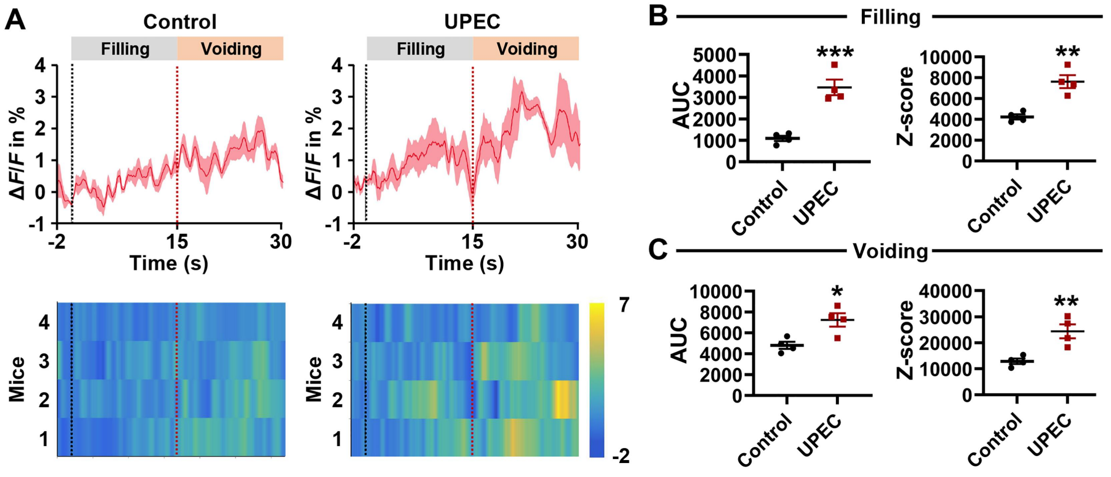

Fig S2. The neural activity of PVN neurons during micturition in mice. (A) Upper: average calcium signals (ΔF/F in %) synchronized during bladder filling and voiding phases in Control and UPEC-infected mice. Thick lines, mean; shaded areas, SEM. Lower: heatmap of calcium signals in each mouse. (B) The AUC and Z-score of calcium signals recording in the PVN neurons during bladder filling phase (n = 4 mice per group). $^{**}P < 0.01$, $^{***}P < 0.001$ vs Control group. (C) The AUC and Z-score of calcium signals recording in the PVN neurons during voiding phase (n = 4

mice per group). *P < 0.05, **P < 0.01 vs Control group. All results are presented as mean ± SEM and analyzed by 2-tailed Student's t-tests.

Supplementary Tables

Table S1. Primers used for quantitative real-time PCR reactions.

**Gene symbol**	**Primer (5'→3')**
GAPDH	Forward: TGTCTCCTGCGACTTCAACA<br>Reverse: GGTGGTCCAGGGTTTCTTACT
Il1β	Forward: GTGTCTTTCCCGTGGACCTT<br>Reverse: AATGGGAACGTCACACACA
Il6	Forward: CTTCTTGGGACTGATGCTGGT<br>Reverse: CTCTGTGAAGTCTCCTCTCCG
Tnfα	Forward: CGGGCAGGTCTACTTTGGAG<br>Reverse: ACCCTGAGCCATAATCCCCT
Il5	Forward: GACAAGCAATGAGACGATGAGG<br>Reverse: CCCACGGACAGTTTGATTCTTC
Il13	Forward: CTTGCTTGCCTTGGTGGTCTC<br>Reverse: GGGAGTCTGGTCTTGTGTGATGT
Csf2	Forward: GCAATTTCACCAAACTCAAGG<br>Reverse: CTCAT TACGCAGGCACAAAAG
Calca	Forward: AAGGGAGCACGTGT TATGGT<br>Reverse: TCCATTCTGAATTGAGGGTGGG
